# A Curious Title

**Published:** 1861-07

**Authors:** 


A CURIOUS TITLE.—A medical book is occasionally issued with a very curious title. One has recently appeared in London, with the following: "The Stomach and its Ailments Practically Arranged; to which *is* added, The Intestinal Canal and its Obstructions." It would be interesting to see an author arranging the stomach practically, and then to add to it deliberately the intestinal tube and its obstructions! This, however, seems to have been successfully done by Dr. Oke, Senior Physician to the South Hants Infirmary, London. We congratulate the gentleman upon his feat. Nothing of the kind, we believe, has yet been achieved on this side of the Atlantic.

